# Gender differences in sexual and reproductive health education in the family: a mixed methods study on Romanian young people

**DOI:** 10.1186/s12889-019-7321-0

**Published:** 2019-08-14

**Authors:** Cristina Faludi, Cornelia Rada

**Affiliations:** 1 Department of Social Work, Faculty of Sociology and Social Work, “Babeș-Bolyai” University, 21 December 1989 Avenue, No. 128, 400604 Cluj-Napoca, Romania; 2 Biomedical Department, “Francisc I. Rainer” Anthropology Institute of the Romanian Academy, Academy House, 13 September Avenue, No.13, 5th District, 050711 Bucharest, Romania

**Keywords:** Sexual education in family, Sexual debut, Romanian youth, Gender differences

## Abstract

**Background:**

The family is one of the key factors that can contribute to reducing the negative consequences of high-risk sexual behavior. This study examines the influence of parents’ communication with children on issues of sexuality on sexual behavior.

**Methods:**

The study is based on a mixed research design. In 2013–2014, 1,359 people aged 18–30 years were randomly selected from urban areas covering the main university centers of Romania, and they completed a questionnaire with 60 items regarding sociodemographic data, family, sexual behavior and health risks. Out of the initial sample, 60 participants agreed to participate in face-to-face interviews, using a thematic interview guide. The quantitative data were analyzed using descriptive inferential statistics, including binary logistic regression. The qualitative data were investigated using thematic analysis.

**Results:**

Exploring the issues of sexual and reproductive health (SRH) discussed with parents according to gender revealed that there was a greater concern in families to address issues of sexuality with girls. The manifestation of any form of sex education in the family was positively associated with a healthy sexual debut, both for women and men (χ2 = 7.759, χ2 = 7.866, *p* = 0.005). The results of the regression reinforced the idea that a lack of sex education in the family decreased the likelihood of a healthy sexual debut, both in women (OR: 0.668, *p* = 0.018) and in men (OR: 0.605, *p* = 0.013). In men, receiving information about sex at a younger age (OR: 0.335, *p* = 0.001) reduced the chance of a healthy sexual debut. Younger women and men were more likely than older women and men to experience a healthy sexual debut [odds ratio (OR): 1.861, *p* < 0.001 and OR: 1.644, *p* = 0.015, respectively]. Qualitative results revealed that SRH talks were generally initiated by young people, usually involved a parent of the same gender and often occurred after events in the sexual lives of young people (after first menstruation/after sexual debut).

**Conclusions:**

In designing health programs for adolescents and youth, the family should be involved in sex education. Modeling family sex education by gender can produce differentiated effects on the sexual debut of men and women.

## Background

Three decades after the change in the aggressive pronatalist politics of Ceausescu’s communist regime, sexual and reproductive health (SRH) problems are far from being resolved. Young people are the most affected because the way they start their sexual life influences their future from the point of view of having a family and children. For example, in 2015, Romania recorded values above the average of EU countries in terms of the number of abortions among women under 20 years per 1,000 live births (374 compared with 289) and the proportion of all live births to mothers aged under 20 years (9.8% compared with 5.1%) [1].

SRH issues can be improved through education and prevention activities undertaken by two main actors: the family and the school. This paper focuses on the role of the family in promoting SRH among young people.

Promoting healthy sexual behaviors during adolescence and early youth is considered to be the most effective way to reduce risk behaviors and decrease the medical costs and health consequences of sexually transmitted infections (STIs) [2, 3]. The interactions between parents and children mark a lasting interpersonal relationship that has a significant and lasting influence on the life of adolescents and young people [4, 5].

Although their friends’ behavior plays an important role in adolescents’ decision to begin a sexual life, parents remain an important source of information about sexuality. Moreover, teenagers are influenced by parents’ attitudes and behaviors [6, 7]. Positive parental role modeling, parental monitoring, perceived connectivity and communication in general are positively associated with late sexual initiation, less intense sexual activity and fewer teenage pregnancies [8].

Girls are involved in conversations about SRH to a greater extent than boys [9]. In regard to communication with girls, parents’ messages about SRH tend to be more cautious and focused on the negative effects of sexual activity [10].

Most messages concerning SRH include protected sex and, in the case of young women, the delay of sexual debut. Parents’ messages to young men and women have been qualitatively differentiated and have been demonstrated to reflect stereotypes about gender roles, sexual desire and the right circumstances for having sex. At the same time, they show a lack of support of young people’s interests in nonheterosexual orientations [9]. Although mothers seem to be the main sex educators in the family, it was found that fathers also play an important role in the sexual socialization of children. Communication between fathers and daughters that is meant to prepare daughters for sexual and romantic encounters can lead to the postponement of sexual debut and a decrease in the frequency of engaging in casual sex [11].

Most studies only investigate conversations about sex/sexuality in general, with questions such as, ‘Have you ever talked about sex?’ Consequently, they offer little information to guide parents on the specific needs in discussions about sexual health [12].

Despite evidence that parents influence adolescents’ sexual behavior, less is known about the specific communications on issues of sexual health in Romanian families.

Given all these considerations, this study aims to investigate the influence of sex education received in the family of origin (FOO) on how Romanian young people initiate their sexual life. The objectives of the quantitative research were i) to investigate gender differences regarding the topics related to SRH issues discussed with the parents; ii) to explore the associations between the content and frequency of discussions in the field of the SRH in the FOO and the age of receiving the first information relating to sex, on the one hand, and the type of sexual debut (healthy versus unhealthy), on the other hand; and iii) to identify the main factors that are associated with a healthy sexual debut. The qualitative component of the study, implemented after the administration of the questionnaire, had as its main objective iv) completing and deepening the perceptions of young people about family discussions on various SRH issues and identifying their own experiences regarding the first sexual intercourse.

## Methods

### Study design

The research underlying this article was based on a two-stage, mixed methodological design. In the initial phase, a cross-sectional quantitative study was carried out, consisting of the administration of an *Omnibus*-type questionnaire. The quantitative cross-sectional study was carried out between 2013 and 2014.

At the time of administering the questionnaire, each subject was asked to complete a written agreement to further participate in a face-to-face in-depth interview concerning family discussions of sexuality and personal experiences of first sexual intercourse. After the end of the quantitative stage of the study, the subjects who agreed to participate to the in-depth interview were contacted, and the qualitative stage ended in the summer of 2016.

### Participants and data collection

In the first stage – quantitative – of the research the subjects were randomly selected from urban areas covering the main university centers of Romania: Timișoara, Zalău, Baia Mare, Cluj-Napoca, Târgu Mureș, Sibiu, Braşov, Piteşti, Craiova, Iaşi, Tulcea, Constanţa, and Bucharest (the capital of Romania). The total number of questionnaires collected in each of the abovementioned towns ranged from 86 to 122, and the number varied according to the number of inhabitants. The subjects resided in these cities, where they also studied or worked, or in large cities in the same geographical area. This research did not involve individuals with known homosexual or bisexual orientation, etc.

In each locality, 2–3 people, with expertise in the fields of sociology, psychology, and medicine, were in charge of organizing, collecting, and verifying the data. Verification of the completion of the questionnaire was done face-to-face with each respondent. The response rate was 100%.

After data cleaning, 1,359 valid questionnaires were obtained. The participants were aged between 18 and 30 years. Subjects who attended an educational institution (preuniversity, university, or postuniversity) completed the questionnaires in class (74.6%). The other participants were employees recruited from educational or cultural institutions or businesses, and they completed the questionnaires at home.

For the quantitative analysis, we selected only the participants who were sexually active. Consequently, our analysis was applied to a sample of 1,164 participants.

In the second stage of the research, participants who agreed to participate in a face-to-face in-depth interview were contacted and included in the study. A thematic interview guide was used concerning issues related to family discussions about sexuality and personal experiences of sexual debut. Thus, 60 young people of the original sample were interviewed.

### Ethical considerations

To ensure that the respondents felt at ease and to guarantee accurate answers, the person who was responsible for checking the questionnaire was of the same gender and as close in age as possible to the respondent. Care and sensitivity were applied all times when dealing with the respondents.

Informed written consent was obtained from each participant at the time of recruitment. The study was approved by the Ethics Commission of the ‘Francisc I. Rainer’ Anthropology Institute of the Romanian Academy, Certificate of Ethical Approval number 285, on the 8th of May 2013.

### Variables and measurements

An *Omnibus*-type questionnaire with 60 items was used to collect sociodemographic data, relevant information about family and items to evaluate health risk behaviors: smoking, alcohol abuse, unprotected sex, sedentary lifestyle, unhealthy eating and violence.

The present study focuses on the relationship between the sex education provided in the FOO and the debut of sexual activity. This aspect has rarely been explored by internal or external studies on the sexual behavior of Romanian young people.

The survey questionnaire included self-reported subjects related to sexuality that were discussed in the familial context. They were assessed based on participants’ answers to the following question: ‘To what extent have you discussed the following subjects with your parents?’ Seven issues were investigated: a) sexual abstinence before marriage; b) menstruation/spontaneous ejaculation; c) how pregnancy occurs; d) prevention of pregnancy; e) abortion; f) sexually transmitted infections (STIs); and g) other sexual problems. A three-point Likert scale was used in the questionnaire. The scale for talking with parents about sex and sexual health proved to have good internal consistency (Cronbach’s Alpha = 0.906). All the items correlated to a good degree with the total scale (lowest *r* = 0.508). The answers from the three-point scale were transformed into binary: a) a lot and b) a little or almost never.

Another variable was composed based on the seven questions. The variable refers to ‘any form of sexual education in the family’ and was recorded as a dichotomized value: a) yes (the respondent had discussed at least one topic related to sexual behavior) and b) no (the absence of any discussion).

The question, ‘What age were you when you first received information on matters of sex?’ was also considered. The answers were structured into three classes: a) 5–11 years, b) 12–14, and c) 15–25 years.

Participants were asked about their age at menarche or first spontaneous ejaculation (FSE). These important events, indicating biological sexual maturation, were recorded as discrete variables.

In this study, the debut of sexual activity means the first sexual intercourse with vaginal penetration. Two intervals of time were constructed, delimiting an early versus a late sexual debut: a) 9–16 and b) 17–26 years. The age of 17 was chosen as a benchmark for a healthy sexual debut, as, in Romania, the majority of youth believe that they have to lose their virginity before finishing their high school studies at the age of 18 or 19. The duration of the relationship, from the beginning of the partnership until the FSI (first sexual intercourse), was captured by the following question: ‘When you first had intercourse, for how long had you known that person (with whom you had your FSI)?’ The answers were categorized as a dichotomized value: a) for less than a month and b) for longer than a month. Additionally, protection at sexual debut was investigated as an answer to the following question: ‘Was the FSI protected?’ The response categories were a) no and b) yes. For those responding affirmatively, an open-ended question followed: ‘How did you protect yourself?’

In terms of qualitative research, the interview guide applied to the young people in the face-to-face discussion included several thematic units, such as a) a specification of the people in the family with whom they had discussed their first menstruation (in the case of girls) or first spontaneous ejaculation (in the case of boys) and preventing pregnancies, of the age at which the discussions took place, of the person who initiated such discussions and of the circumstances that generated the discussions; b) a description of the advice offered by their parents regarding the beginning of their sexual lives; c) a reflection on the impact of sexual education received from both mothers and fathers on their attitudes concerning their sexual debut or their experiences of first sexual intercourse (for young people who had already started their sexual lives); d) a description of their experiences of first sexual intercourse (the place where it happened, the type of relationship with the sexual partner, the method of protection, the emotional impact of this experience); and e) a specification of their needs regarding sexual education and which topics should be covered by parents (the content of sexual education, the age at which the discussions should begin, the state of comfort between parents and children during the discussion of sensitive issues).

### Data analysis

According to the objectives of the quantitative research, inferential statistics (Pearson’s chi-square test and independent-samples T test) and multivariate analysis (binary logistic regression) were applied. The data were analyzed with the SPSS 19 program.

In the context of the inferential analysis, we tested to what extent there is a link between discussing topics on SRH themes in the FOO and the age at which young people received their first information on sex, on one hand, and a healthy sexual debut, on the other.

A composite variable of healthy sexual debut was built that involved the simultaneous fulfillment of the following three conditions: age 17 or older, use of a condom as protection at the FSI, and being in a couple relationship for over 1 month.

To investigate the most important factors that may influence the context in which young people start their sexual lives, three logistic regression models were conceived: one for the entire sample, one for men, and one for women. The binary dependent variable is represented by the type of sexual debut, where a value of 1 means a healthy sexual debut and 0 means a high-risk sexual debut in terms of SRH.

Explanatory variables were entered into the regression model for the entire sample: gender, age, age range at the time of receiving their first information on sex, and any form of sex education in the FOO. Statistical analyses were performed on two age groups: 18–22 years and 23–30 years. The 18–23 age range marks the completion of university studies, the search for a stable job and the development of intimacy and a couple relationship, while the 24–35 age group prioritizes an investment in career and the foundation of a family/a stable partnership, possibly including childbearing and rearing.

Regarding the qualitative data, thematic analysis was used, following the main thematic units included in the interview guide. After the qualitative analysis of the data, we selected responses that would add more knowledge and understanding regarding the objectives of the qualitative research.

## Results

### Descriptive analysis results

Table 1 displays the basic structure of the sample and the characteristics of sexual debut. Out of total number of the participants in the study, over half were women and at the time of the survey were aged between 23 and 30 years. More than three-quarters of the young people in the sample started their sexual life at the age of 17 and at the time of the onset of sexual life had been having a relationship longer than 1 month. At the first sexual intercourse, 65% of the young people used the condom. More than half of the participants obtained the first information about sexuality when they were 12–14 years old.

**Table 1:** Main characteristics of the participants

Characteristic/ variable	N	%
Gender		
Female	665	57.1
Male	449	42.9
Age group (years)		
18–22	569	48.9
23–30	595	51.1
Age at first sexual intercourse (years)		
9–16	290	24.9
17–26	874	75.1
Duration of the current consensual union (until first intercourse)		
Less than a month	153	13.1
More than a month	1011	86.9
Use of condom (pill) at first intercourse		
Yes	755	64.9
No	409	35.1
Age at receiving first information about sexuality (years)		
5–11	188	16.2
12–14	661	56.8
15–25	315	27.1

Table 2 presents the distribution of participants, irrespective of gender, according to the main topics related to SRH discussed with parents. The most discussed topics in the family were, in decreasing order, discussions about pregnancy prevention, menstruation/ FSE and STIs. The less approached subjects in the discussions with their parents were abstinence before marriage (16.8%) and abortion (24%). Over half of the participants discussed intensively at least one of the seven investigated topics (56.4%) and under 10% discussed in depth about all seven topics.

**Table 2:** Distribution of participants according to the main topics related to SRH discussed with parents

Discussion with parents about	A lot	Little or almost nothing
	%	
Sexual abstinence before marriage	16.8	83.2
Menstruation/ spontaneous ejaculation	37.4	62.6
How pregnancy occurs	32.6	67.4
Prevention of pregnancy	39.0	61.0
Abortion	24.0	76.0
Sexually transmitted infections (STIs)	34.8	65.2
Any sexual problem	27.3	72.7
All seven topics	8.2	91.8
	Yes	No
Any topic of sexuality discussed with parents	56.4	43.6

**Fig. 1:**
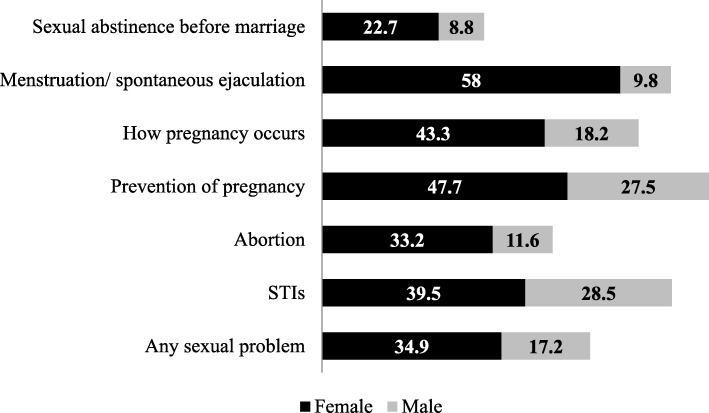
The subjects discussed in the FOO regarding SRH by gender

Regarding the hierarchy of which subjects related to SRH were most commonly approached in the family, the first place belonged to the discussions about menstruation (58%) in women and sexually transmitted infections (28.5%) in men. Discussions dedicated to the prevention of pregnancies (47.7 and 27.5% in women and men, respectively) came in second place for both genders. Discussions about how pregnancy occurs (43.3 and 18.2% in women and men, respectively) were in third place (Fig. 1).

Perhaps the most relevant finding concerning family discussions on sexual and reproductive issues was that for each of the health topics investigated in the present study, the frequency with which such discussions were held was much higher in the case of women than in the case of men.

Thus, close examination of the results in Fig. 1 reveals that more than half of the women reported that they had benefited from discussions on the topic of menstruation, while the discussion on spontaneous ejaculation occurred with a 6-fold lower frequency in men. The parents of girls discussed abortion and how pregnancy occurs more than twice as frequently as the parents of boys. The least popular subject among both women and men was related to sexual abstinence before marriage; however, in this case, women also reported discussing this behavior to 2.6 times more frequently than men (Fig. 1).

**Fig. 2:**
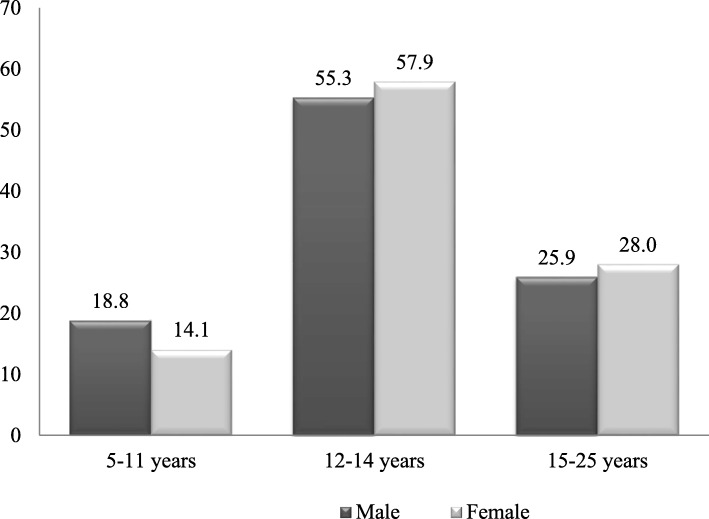
The age at which young people first received information on sex by gender

In the analyzed sample, slight gender differences were noticed regarding the age at which young people first received information on sex. More men than women received their first information about sexuality at young ages ranging between 5 and 11 years (18.8% versus 14.1%) (Fig. 2).

However, men experienced their first ejaculation later than women experienced menarche, and they were less likely than women to know the age at which this first event of sexual maturation took place. Thus, the response rate was 100% in females and 62.9% in males. The analysis of the answers showed that 81.6% of women had their menarche and 64.1% of men had their FSE between 12 and 14 years old, and the average age at the occurrence of the event was 13 years for women and 14 years for men. These factual data are relevant for a better understanding of the context in which family discussions on SRH issues took place, as they will be discussed in the qualitative data analysis section.

### Inferential analysis results

The analyzed sample revealed that girls showed more protective behavior than boys at first sexual intercourse. More than half of the girls and less than one third of the boys had a healthy sexual debut (55.5% versus 30.5%, χ^2^ = 72.22, *p* < 0.001, data not shown in Table 3).

**Table 3:** Relationships between the studied variables and the healthy / unhealthy sexual debut

Study variables	Women			Men		
	Healthy sexual debut					
	Yes (%)	No (%)	Indicator / (p)	Yes (%)	No (%)	Indicator / (p)
Any topic of sexuality discussed in the family						
Yes	59.2	40.8	χ^2^ = 7.759	37.4	62.6	χ^2^ = 7.866
No	47.6	52.4	(0.005)	25.7	74.3	(0.005)
Mean age at first information about sexuality	13.46	13.56	t = 0.611 (ns)	13.63	12.93	t = 3.219 (0.001)
	Dif. average values = − 0.106			Dif. average values = 0.704		
The level of discussion about sexual matters: Sexual abstinence before marriage						
A lot	55.6	44.4)	χ^2^ = 0.002	43.2	56.8)	χ^2^ = 3.686
Little or almost nothing	55.4	44.6	(ns)	29.2	70.8)	(0.055)
Menstruation/spontaneous ejaculation						
A lot	59.8	40.2	χ^2^ = 7.068	42.9	57.1	χ^2^ = 3.942
Little or almost nothing	49.5	50.5	(0.008)	29.1	70.9	(0.047)
Prevention of pregnancy						
A lot	57.4	42.6	χ^2^ = 0.908	39.4	60.6	χ^2^ = 7.150
Little or almost nothing	53.7	46.3	(ns)	27.1	72.9	(0.007)
STIs						
A lot	60.8	39.2	χ^2^ = 5.038	34.5	65.5	χ^2^ = 1.534
Little or almost nothing	52.0	48.0	(0.025)	28.9	71.1	(ns)

Note: *p* significance level, *ns* not significant

According to Table 3, the manifestation of any form of sex education in the family was positively associated with a healthy sexual debut, both for women and men (χ^2^ = 7.759, χ^2^ = 7.866, *p* = 0.005). It was noted that among women, a positive association was maintained between the approach of each subject and a healthy sexual debut, but the results were significant only for the discussion on menstruation (χ^2^ = 7.068, *p* = 0.008) and on STIs (χ^2^ = 5.038, *p* = 0.025). Among men, the most powerful significant positive association was registered in the discussion about pregnancy prevention (χ^2^ = 7.150, *p* = 0.007).

Regarding the average age of first receiving information on sex, an older age was associated with a healthy sexual debut among men (t = 3.219, *p* = 0.001). Instead, women who had received their first information on sexuality earlier were more likely to experience a healthier sexual debut than those who received this information later, but the age difference was negligible (Table 3).

### Logistic regression results

The results of the three logistic regression models are shown in Table 4. The model for the entire sample reflected that the profile of those who experienced a healthy sexual debut exhibits the following characteristics: women aged 18 to 22 years who received their first information on sexual matters between 15 and 25 years old and who benefitted from any kind of sex education in the family.

**Table 4:** Results of logistic regression regarding a healthy sexual debut

Type of variables	All OR (p)	Women OR (p)	Men OR (p)
Control variables			
Gender			
Female (ref.)	1		
Male	0.415 (< 0.001)		
Age group			
23–30 (ref.)	1	1	1
18–22	1.765 (< 0.001)	1.861 (< 0.001)	1.644 (0.015)
Age at first information about sex			
15–25 (ref.)	1	1	1
5–11	0.572 (0.005)	0.822 (ns)	0.335 (0.001)
12–14	0.893 (ns)	1.073 (ns)	0.669 (0.079)
Any sexual information in the family			
Yes (ref.)	1	1	1
No	0.637 (0.001)	0.668 (0.018)	0.605 (0.013)

Note: *OR* odds ratios, *p* significance level, *ns* not significant

In the subsample of women, the logistic regression results indicate that young women in the 18-to-22-year-old age group were almost twice as likely to experience a healthy sexual debut compared to those who were between 23 and 30 years old at the time of the study [odds ratio (OR): 1.861, *p* < 0.001]. Women who had not received any form of sex education from their parents were nearly half as likely to experience a healthy sexual debut (OR: 0.668, *p* = 0.018).

For the subsample of men, the effect of age and sex education in the FOO was the same as for women.

While among women, the context of sexual debut was not influenced by the age at which they acquired their first information about sex, among men, the younger they were when they acquired their first information on sex, the lower their probability was of experiencing a healthy sexual debut (OR: 0.669, *p* = 0.079, at 12–14 years; OR: 0.335, *p* = 0.001 at 5–11 years) (Table 4).

### Qualitative results

The questionnaire did not distinguish between parents (mother or father) regarding discussion of SRH topics in the FOO. In addition, the questionnaire did not ask who initiated the conversation on SRH topics: the parent or the child. Additionally, it did not ask if discussions about sexuality were held before or after the FSI or if they were a cause or an effect of the sexual debut.

In contrast, the interview guide did allow the investigation of these issues. In line with the main objective of the qualitative research and the results obtained from the application of the questionnaire, the qualitative data analysis focused on the context in which the main topics of SRH were addressed within the family and how these discussions occurred in relation to the experience of first sexual intercourse in the case of young people. With emphasis on SRH issues, other sexual education actors outside the family, such as the school, friends, and acquaintances, were included in the qualitative analysis.

Among the 60 conducted interviewed participants, 35 women who had begun their sexual lives were identified. The age at which these women first obtained information about menstruation was 10–11 years old, and the most common age at menarche was 12 years old. Almost a third of the women had received information about menstruation at the time of or after menarche, and a quarter of the women had received it a year before their first menstruation. Most women received information about the menstrual cycle from their mothers, or the discussions with their mothers were preceded by conversations with other women in the family (an elder sister, aunt, grandmother) or with people at school (classmates, a teacher, or a biology teacher). Some women first learned about menstruation from older friends who had already experienced it. In over half of the cases, the discussion on the menstrual cycle was initiated by the daughter.

*I found out from a friend about menstruation as she had already got her first menstruation; then I asked my mother... (Although) I am not comfortable talking about this subject (because) my parents are not very open on this issue. (Yet) mother gave me the necessary answers and information. She said that, from that moment, I had turned from a little girl into a teenager and I have to be careful and take care of myself* (Female 1, 20 years old, menarche at 12, first information about menstruation at 11, and sexual debut at 19 years old).

In regard to the 11 surveyed men who had started their sexual lives, more than half had received their first information about FSE at 12 years old, which was approximately 1 year later than the most common age at which women received their first information about menstruation. Over a third of the men reported that they had their FSE a year after receiving preliminary information about FSE. In three cases, the men did not remember the age of their first FSE. Most commonly, men had obtained their first information about FSE from friends in their neighborhoods or from older friends. Other sources of information were school, the Internet, the family physician and, only in one case, the father. More than half of the men had not approached this topic with their parents.

*At the age of 15, I had, for the first time, a discussion with a parent about male puberty, called FSE. I had that conversation with my father when I experienced that puberty problem because I had become a little scared of that problem, even though I had talked to a family physician before... The contribution of my father to my sex education consisted of his advice and examples and of his way of being open to such discussions. My mother contributed to my sex education with advice and even with examples offered to my father during my discussions with him* (Male 1, 19 years old, first FSE at 15, first information about FSE at 15, sexual debut at 17 years old).

Over a third of the surveyed women began their sexual life and received their first information about preventing pregnancy at the age of 14. Schools were the first source of information about pregnancy prevention for half of the interviewed women. The discussion about pregnancy prevention was most frequently approached by teachers (the biology teacher and/or a class teacher), by members of the medical staff (the school nurse or doctor, a gynecologist or a sex education class given by a physician), and by representatives of NGOs and companies with the aim of preventing pregnancies and STIs and promoting sexual hygiene in schools (e.g., Always brand). In over a third of the cases, mothers were the main source of information concerning pregnancy prevention. In only two cases, women stated that the source of information on this subject was the Internet. It is worrying that over a quarter of the women had never talked with their parents about pregnancy prevention. Only in four cases had the women discussed this issue with both parents.

*I hope I’m recalling correctly that my first conversation of this kind was with the female doctor at school. I remember when she came with a poster, and we were all fascinated by what she was telling us... At that time in my childhood (age 13), I was not very interested in that subject, but, entering adolescence, everything seemed to become clearer. Mom, the most honest person I know, has always given me examples of her mistakes in her youth. When I was 14-15 years old, I found out my mother’s painful secret – that she had had an abortion. I had conversations with my father about that subject only after I had started my sexual life. I had all the necessary information, all my mother’s gentle advice, and yet I did not listen to her and I started my sexual life at a time when I was not ready mentally... Mom helped in that I always knew how important protection and the assumption of responsibility are* (Female 2, 21 years old, first information about pregnancy prevention at 13, and sexual debut at 16 years old).

Regarding their first information about pregnancy prevention, most (nearly half) of the men received it a year later than most of the surveyed women, i.e., at 15 years old. The primary sources of information on pregnancy prevention were schools and parents (four cases each); one man mentioned his friends as a source of information, and another mentioned the Internet. However, half of the interviewed men had not approached this topic with their parents.

*The conversation about pregnancy prevention occurred when my parents found out that I, at the age of 16, was in a relation with a girl who was three years older than me. This conversation was mostly with my father because I refused to talk to my mother because of the embarrassment surrounding such a topic. My parents’ advice regarded methods of avoiding pregnancy and protection against sexually transmitted diseases... I find it easy to talk with my parents about intimate aspects of my life, especially related to sex, because (discussions) are aimed at my education and guidance to a controlled sexual life* (Male 1, 19 years old, first information about pregnancy prevention at 16, and sexual debut at 17 years old).

Among the 46 interviews with the subjects who had begun their sexual lives, 41 used a condom during their FSI; in one case, pills were used; and in two cases, protection was not used, as it was the wedding night. Regarding the favorite method of protection, in most cases, the man or both partners were in charge of the decision to use it. Over three-quarters of the young people who had started their sexual lives talked to their partner about contraception before their FSI.

*At my FSI, I used a condom. Both partners should be in charge of protection, and I think it’s impossible for a couple not to discuss methods of protection; so yes, I had talked about it with my partner... I’m in a relationship with the partner with whom I started my sexual life almost two years and a half ago, and the ‘event’ happened after a fairly long period of time because I wanted to make sure that our relationship was not based only on that and, just as importantly, the emotional involvement was very important. It was not something precisely planned, but rather something spontaneous* (Female 3, 19 years old, menarche at 13, first information about menstruation at 9-10, first information about pregnancy prevention at 14, and sexual debut at 17 years old).

*I discussed it with my partner, and we established that I would be in charge of protection and that the chosen method was condoms. I started my sexual life... in the apartment of a friend. She was not a casual partner; I am still in a relationship with her. At the time, I had known her for at least one year, and it was something that we had been thinking about. It was all planned, and we felt then that the time was right. I am still involved with her; we get along very well, and it is possible (that I might have) future plans with her* (Male 2, 24 years old, first information about FSE at 12, first FSE at 14, first information about pregnancy prevention at 12, and sexual debut at 17 years old).

The paragraph below reveals the views of an interviewed young woman on the importance of a sexual life and her need for sex education topics:

*My mother advised me a lot, as she is a nurse. (She advised me) to protect myself, because I can get sexually transmitted diseases, and to begin my sexual life with a man I know and when I feel ready, not casually. A sexually active life is necessary, as it is good for health. Physically, pleasure improves circulation and increases energy. Its emotional effects include greater self-esteem, self-confidence and a lively, more dynamic spirit. Spiritually speaking, pleasure can open the door of happiness and contributes to a happier and more optimistic life approach* (Female 4, 21 years old, menarche at 15, first information about menstruation at 10-11, first information about pregnancy prevention at 15, and sexual debut at 18 years old).

Most of the young people admitted that discussions with their parents about sexual life were difficult because of the embarrassment they felt in revealing intimate experiences: ‘*I am ashamed that she (her mother) is older, and I’m afraid that sometimes she does not understand things. There is no other reason than shame and shyness*’ (Female 5, 21 years old).

When asked about who should be in charge of sex education, most of the respondents answered that parents should ideally be the first to provide education, followed by schools:

*Currently, small children have access to the Internet, where such information can be found very easily. Therefore, I believe that parents should provide sex education before their children find out the wrong information so that they can better understand what sex involves and what its risks are. After having been informed by their parents, children can attend sex education classes in schools, which could offer them a different perspective or more details* (Female 3, 19 years old).

## Discussion

This article has explored the way in which parents’ communication with their children on issues of sexuality and SRH determines when, how and with whom Romanian young people start their sexual lives. The study revealed that the frequency and content of sexual education in the FOO differs significantly by gender. The quantitative results showed that the most discussed subjects in the family of origin were those concerning menstruation in the case of girls and sexually transmitted infections in the case of boys. These were followed, for both sexes, by the discussion concerning pregnancy prevention and how pregnancy occurs. For all SRH topics discussed in the family, the percentages reported for young women are far higher than those reported for young men. The results of inferential analysis have shown that more intensive sex education is closely related to a later sexual debut that involves the use of protection and takes place in the context of a stable relationship. The results of multivariate analysis have shown that young women have adopted more protective behavior at the time of the sexual onset compared to young men. Among both sexes, younger respondents and those who have had discussions about SRH in their family of origin had a higher probability of experiencing a healthy sexual onset. A younger age in finding the first information about sexuality among young men proved to increase the chance of a high-risk sexual onset.

The qualitative results of this study emphasized that within the family, the mother assumes and maintains the discussion of sexual topics in the case of young girls, while the father is less involved in the sexual education of children. To a large extent, the family discussions about various aspects of sexuality occurred near or after the occurrence of sexual life events (e.g. after the onset of sexual life). Although young people confessed that they had felt uncomfortable talking to their parents about their intimate life, they declared that they would have preferred that parents should have been the first to offer them information about sexuality.

This topic of SRH education in the family has recently become of interest to Romanian researchers. Thus, a study applied between 2011 and 2012 on a sample of 1,215 Romanians aged 18–74 years revealed that only 4.2% of participants had discussed all the investigated issues of sexuality and SRH ‘a lot’ with their parents [13], while this study shows that Romanian young people (18–30 years old) discussed all seven topics ‘a lot’ with their parents, at a rate of nearly 2 times higher (8.2%) than that reported in the previous study. This may indicate, on the one hand, that parents of the younger generations are aware of the growing importance of communication about health sexuality issues with their children and, on the other hand, that young people today want to know more about sexuality from their parents. This idea was also suggested by a previous study on Romanian women that reported that students in the first and second year of study said that young people of their age would prefer to obtain their first information about sex from their parents [14].

However, over half of the young people in the sample (56.4%) had discussed at least one of the investigated themes of sexuality ‘a lot’ with their parents. This result is higher than that found in other studies conducted in Zimbabwe (44%) and Ethiopia (36.9%) but lower than that found in a study in Malawi (74%) [15, 16]. In contrast, Thai girls do not usually discuss sexual matters with parents, as the latter fear this might encourage sexual activity [17]. These differences can be explained both by demographic and cultural differences and the differences in accessing SRH information and services.

In this study, 16.8% of the young people discussed sexual abstinence before marriage ‘a lot’ with their parents. The fact that Romanian families still discuss abstinence before marriage indicates that there is still a segment of parents who consider virginity to be a valuable and desirable reproductive behavior in the intimate lives of young women. In addition, sexual abstinence before marriage was a frequent traditional custom in Romania, especially in rural areas. The norms regarding age at the time of first sexual activity, marriage and motherhood have changed. As a result of emancipation, women have become sexually active before marriage and postponed marriage and their first childbirth [18]. The causes are numerous, including the weakening of traditional rules, better education, the modernization of society, the increase in internal migration, and, after 1990, the influence of the media, which is more aggressive and more diversified [19].

The study also revealed that the top three issues of sexuality that were discussed in the family, for both women and men, included pregnancy prevention and pregnancy occurrence. It seems that Romanian families are concerned with the education of their children on the election of the best time, psychologically, socially and financially, to become parents. This concern of the Romanian families has been documented by another Romanian study [13].

The discussion about abortion occurs last in women. This is a surprising result, given the continually high rates of abortion on demand in Romania. This indicates that abortion continues to be an acceptable solution, psychologically, socially, financially and morally, for Romanian women. The study of Frejka [20] on several European countries showed that between 1990 and 2000, Romania recorded the highest rate of abortions. The peak of the phenomenon was observed in the first year after the fall of communism when abortion became legal. Even though modern contraceptives became widely available in the region of Eastern Europe after 1990, Romanian women faced with an unwanted pregnancy continued to turn to abortion as a method of regulating fertility with wide normative acceptance [21].

Most of the young Romanians in this study received their first information related to sex in the 12–14 age range, a period that coincides with the time of menarche or FSE. As in other studies [13], a correlation was found between these events of sexual and reproductive growth and first information on sex.

The results of multivariate analysis showed that younger women more frequently experienced a healthy sexual debut than older women. However, women who received sex education in the family had more opportunities to experience a healthy sexual debut.

Similar to other studies, this study draws attention to the fact that boys are a priority group for sexual health services, as they engage in greater sexual risk behaviors than girls. However, they are less likely to seek sexual health information or access sexual health services [22, 23].

The results of this study have a series of implications for public policy decision makers and SRH professionals. First of all, if we take into account the fact that young people prefer their parents as primary sources of information about their own sexuality, then discussions on this topic should begin when children start to show interest in this area and should be adapted to the age of children, their understanding [24, 25], family values, customs, and the local cultural framework [26].

Secondly, it is not enough to delegate mothers to take care of their daughters’ SRH education. The involvement of both parents in preparing young men and women to understand and accept the physiological and psychological changes specific to sexual maturity would encompass the personality of the young person through a harmonious integration of the sexuality dimension. Boys should be equally educated by both parents in order to adopt – together with the girls – precautions regarding their sexual life.

Thirdly, an effective approach of SRH issues can find the parents unprepared because children can access information about sexuality, with or without their parents’ approval, especially through media and social networks. Therefore, parents need to have knowledge, to acquire appropriate communication skills with their children in this field, and to be able to provide a comfortable, trustworthy framework for such family discussions. Until now, the unsatisfied needs of young Romanians to find out information about SRH from parents and the lack of parents’ knowledge and skills to address these issues are also accompanied by the absence of SRH specialized services and programs. In Romania, there are still no comprehensive education SRH programs in schools, and the specialized social services for young women facing a pregnancy crisis or for couples confronted with the adverse effects of a high-risk sexual life are extremely rare (e.g. maternity centers) and in most cases based on humanitarian aid and on volunteer work (e.g. pro-life centers, called Pro Vita). In the future, things could be improved at the society level, at least in terms of preventing sexual risk behaviors, by joint efforts of parents and teachers to promote SRH programs in schools, on the basis of which young women and men should be able to build a healthy sexual life in adulthood [27, 28].

This study has several strengths. First, it addresses the scarcity of English-language studies that investigate the role of sexual education in the FOO on the sexual and reproductive behavior of young people, with a focus on sexual debut. This study has shown that young people benefit more from family discussions on sexuality compared to earlier generations, which has positive consequences for their sexual debut. Second, the study allowed analysis of the content and frequency of sex education in the family and its influence on sexual behavior by gender. This type of analysis presents an advantage because, until recently, most women were the target of SRH health studies, which excluded the men’s perspective. The obvious differences by gender that were found in this study may be useful in the design of evidence-based SRH programs. Third, the quantitative investigation was combined with a qualitative exploration, which deepened the analysis and enriched the data on sexual education in the FOO that was acquired in the questionnaire.

The current study has some limitations. The quantitative study could not indicate the source of the first sex information that young Romanians received (e.g., family, group of friends, school, media or Internet). This aspect is important in designing SRH education programs, as the preferred sources of information in young people change with age. For example, a Canadian study showed that as children grow, they tend to consult their parents less [22].

Parents’ religious beliefs and values can influence and limit the approached sexual health issues [24]. This aspect was not covered by the quantitative study. In families where parents are more religious and have traditionalist values, the discussion of abstinence tends to precede conversations about topics such as condom use or birth control.

Another limitation is that the sample is not nationally representative. However, it can be considered an adequate representation for young people in a higher education institution who are living with parents and childless.

## Conclusions

This study showed that there was a significant positive association between discussions about sexuality and sexual health, on the one hand, and a healthy sexual debut, on the other hand. The results from the questionnaires, as supplemented by the information from the interviews, showed that a considerable proportion of Romanian youth embraces cautious sexual behavior and holds quite healthy conceptions regarding the appropriate context for effective sexual education in the FOO.

The study has shown that younger generations are increasingly well informed in matters of sex. In addition, given that the young people in the sample lived in urban areas, they had greater access to sources of information and modern contraception than rural youth.

In Romania, the relationship between parents and offspring is more emancipated than it was in previous years, and discussions on sexual matters are no longer a taboo. However, while parents are inclined to discuss menstruation/spontaneous ejaculation, protection at sexual intercourse, and abortion with their children, they are not yet prone to approach subjects such as sexual satisfaction, pleasure, or affective needs that are involved in intimate relationships. Such discussion should not be avoided considering that mass media abound in erroneous information on these sensitive topics.

For all these reasons, parents should be engaged as partners in any program designed to improve the SRH of their children. It is necessary that future interventions help parents, both mothers and fathers, improve their comfort when addressing issues of sexuality with their children to identify barriers to effective communication and to obtain practical skills that are meant to promote delayed sexual debut and safer sex behaviors among adolescents and young people.

## Data Availability

The datasets used and/or analysed during the current study are available to the two authors of the article upon reasonable request.

## Abbreviations

FOO: Family of origin
FSE: First spontaneous ejaculation
FSI: First sexual intercourse
SRH: Sexual and reproductive health
STIs: Sexually transmitted infections
